# Solution NMR reveals micelle-stabilized α-helical segments flanking the RIPK1 RHIM amyloid core

**DOI:** 10.1016/j.bbrc.2026.153513

**Published:** 2026-02-21

**Authors:** Paula Polonio, Gustavo A. Titaux-Delgado, Miguel Mompeán

**Affiliations:** 1 Instituto de Química Física Blas-Cabrera (IQF- CSIC), Madrid, Spain; 2 Universidad Autónoma de Madrid, Escuela de Doctorado, Madrid, Spain

## Abstract

Receptor Interacting Protein Kinase 1 (RIP1 or RIPK1) can transition from a monomeric state to amyloid assemblies during cell signaling via a conserved RIP homotypic interaction motif (RHIM). Through RHIM–RHIM interactions, RIPK1 forms both homomeric and heteromeric amyloids implicated in programmed cell death, but solution-state characterization of unassembled RIPK1 is hindered by rapid aggregation. Building on our recent RIPK1 fibril structure, we designed an aggregation-slowing mutant (N545D) and show that two segments within RIPK1’s disordered domain display nascent helical propensity under near-physiological pH conditions. SDS micelles further stabilize these helices and enable measurements on the wild-type sequence. Notably, analogous conditions do not reveal comparable α-helical populations in RIPK3, despite a conserved RHIM sequence and amyloid structure endpoint.

## Introduction

Receptor Interacting Protein Kinase 1 (RIPK1) is a pivotal regulator of cellular fate in mammals. Its “Janus face” reflects a dual role in orchestrating intracellular pathways controlling cell death, survival, and inflammation through regulated signaling assemblies. RIPK1 comprises two structurally well-characterized domains: an N-terminal kinase domain (1) and a C-terminal death domain (2). Between these regions lies a long interdomain segment that includes ~20 residues defined as the RIP homotypic interaction motif (RHIM). This motif is characterized by a conserved tetrad with an (I/V)-Q-(I/V/L/C)-G consensus sequence prone to amyloid assembly. In RIPK1, this tetrad corresponds to the sequence IQIG. Similar RHIM motifs are also found in other RIPK family members (e.g., RIPK3) and in additional cell-fate regulators, where RHIM–RHIM interactions are essential for the formation of both homomeric and heteromeric amyloid-like signaling assemblies (3–8).

Recently, structural insights into RHIM-mediated amyloid assemblies have emerged from solid-state NMR (SSNMR) and cryo-electron microscopy (cryo-EM) studies (9–13). In particular, assemblies formed by the RIPK1 RHIM have been shown to adopt a core cross-β architecture stabilized by critical side chain–backbone hydrogen bonds, with residue N545 identified as a key determinant of RIPK1–RIPK3 heteromerization (12). While these amyloid structures illuminate the assembled state, they do not directly report on the conformational tendencies of the RHIM in solution prior to assembly. Experimentally, this question is compounded by the strong propensity of RIPK1 to aggregate rapidly into amyloid fibrils, precluding solution-state characterization of unassembled monomers. Whether and how the RIPK1 RHIM samples nascent conformations in its monomeric state, and how such tendencies may influence nucleation or regulation of amyloid formation, therefore remains an open question.

To access these pre-assembly states, an approach is needed that preserves the RHIM sequence context while mitigating rapid aggregation. Guided by our recent RIPK1 amyloid structure and the central role of N545 (12), we used the previously described slow-aggregating N545D variant to enable solution NMR characterization of the unassembled ensemble. In parallel, disorder-informed sequence analysis and structure prediction can guide experimental interrogation of latent secondary-structure propensities. Because such biases can be subtle in aqueous solution, complementary conditions that stabilize transient helices while suppressing aggregation are often needed to extend measurements to the wild-type sequence.

Micellar systems offer a practical means to stabilize transient conformations and suppress aggregation at near-physiological pH, enabling solution-state examination of otherwise short-lived ensembles. Several studies have shown that micelles can favor helical conformations in intrinsically disordered proteins (IDPs) and amyloid-forming proteins (14–17). Notably, the RHIM region of RIPK3 was recently reported to interact with membrane mimetics, with SDS micelles acting as effective inhibitors of RIPK3 aggregation while facilitating the detection of nascent structural features at near-physiological pH (18). Here, we leverage this concept in a structure-guided workflow: we first use solution NMR to characterize a slow-aggregating RIPK1 variant under aqueous, near-physiological conditions, and then employ SDS micelles as a stabilizing, aggregation-suppressing environment to amplify subtle helical propensities and extend measurements to the wild-type sequence. This framework also enables direct comparison to RIPK3 under analogous micellar conditions, allowing us to assess whether conserved RHIM amyloid endpoints arise from similar or distinct pre-assembly conformational biases.

## Results

### Rapid aggregation at near-physiological pH prevents solution-state characterization of RIPK1 RHIM

**Figure 1:**
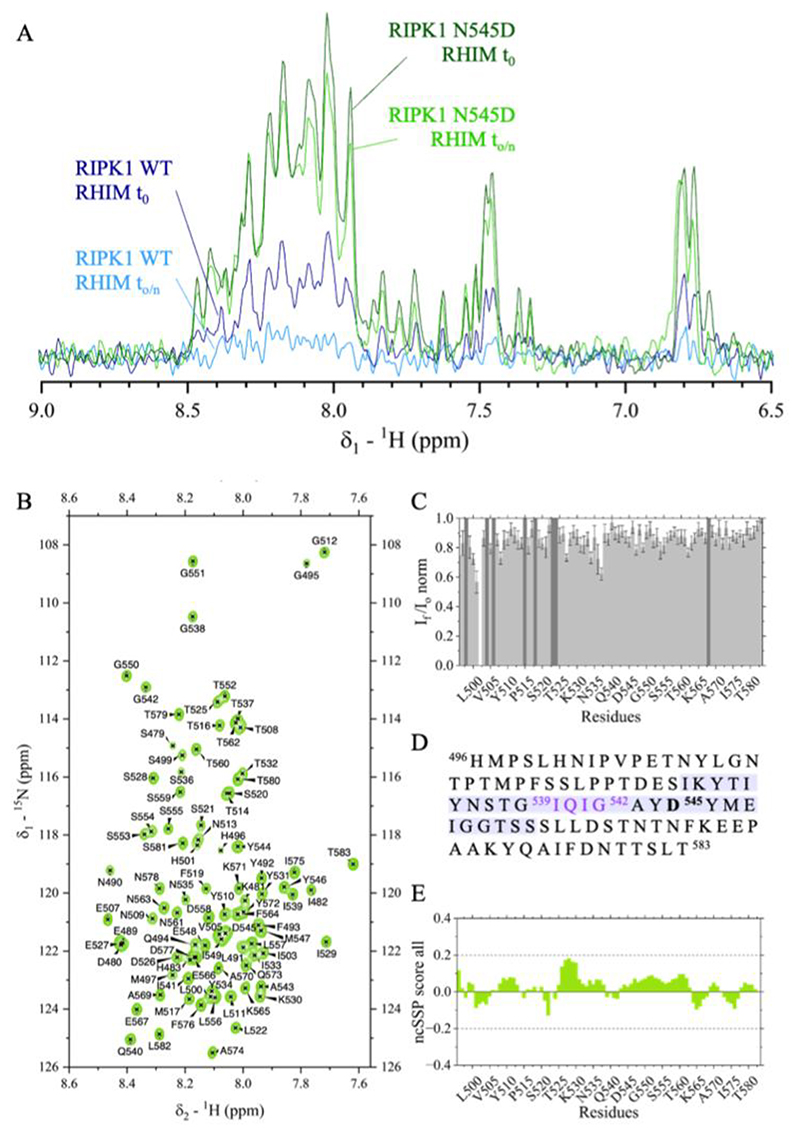
RIPK1 RHIM in 20 mM MES, pH 6.6. (**A**) First FID from ^1^H,^15^N-HSQC of RIPK1 RHIM WT (blue) and N545D (green) recorded immediately after preparation (t0) and after overnight incubation (o/n). **(B)** Assigned ^1^H,^15^N-HSQC. **(C)** Normalized peak-intensity decay over 3 h (prolines in dark grey). **(D)** Construct sequence (496–583); fibril core (light purple), RHIM tetrad (lavender), mutation (bold). **(E)** ncSSP profile; dashed lines indicate the ±0.2 threshold for nascent structure.

Building on previous studies of RHIM proteins in solution (19), we attempted to access pre-assembly information by exchanging RIPK1 RHIM from denaturing conditions into a near-physiological buffer. Uniformly ^13^C,^15^N-labeled protein was purified in 8 M urea (pH 8.0) and exchanged into 20 mM MES, 5% D2O, pH 6.6. Even at <10 μM, samples showed rapid NMR signal loss, with no detectable signals after overnight incubation (Figure 1A).

Recent structural studies of RIPK1 RHIM fibrils revealed key hydrogen bonding interactions critical for assembly stability (12). Notably, substitution of residue N545 by an acidic residue was previously reported to inhibit RIPK1 heteromerization with RIPK3 (3), and the fibril structure showed that N545 regulates both homomeric nucleation and heteromeric stabilization via a side chain-to-backbone hydrogen bond (12). Based on these findings, we hypothesized that the N545D mutation, which delays fibril formation, could provide a sufficiently long solution-state NMR window to characterize the RHIM prior to assembly, and thus allow assessment of the core IQIG tetrad in amyloid formation.

Following the same procedure used for RIPK1 WT RHIM, a His-tagged construct encompassing residues 496–583 carrying the N545D mutation was produced as isotopically ^13^C,^15^N-labelled protein and purified under denaturing conditions. Buffer exchange into 20 mM MES, 5% D2O, pH 6.6 yielded a final protein concentration of ~21 μM. In contrast to the WT construct, the sample showed minimal aggregation and remained largely soluble overnight (Figure 1A), enabling 3D NMR experiments without significant signal loss.

### Solution NMR of a slow-aggregating variant reveals a largely disordered ensemble with subtle helical tendencies

Based on the delayed aggregation of the N545D variant, we characterized RIPK1 RHIM at near-physiological pH using solution NMR. Using amide–amide and backbone correlation experiments, we assigned 77 out of 78 non-proline residues in the ^1^H,^15^N-HSQC spectrum, covering 99% of Cα and 81% of Cβ resonances (Figure 1B). Intensity decay during 3D acquisition is shown in Figure 1C. The sequence is shown in Figure 1D, highlighting the IQIG tetrad and, in light purple, residues forming the amyloid fibril core.

From experimental chemical shifts, we calculated neighbor-corrected secondary structure propensity (ncSSP) scores using the reference library of Tamiola et al. (20). The ncSSP profile revealed an overall absence of stable and highly populated secondary structure (Figure 1E), qualitatively resembling that reported for RIPK3 under non-aggregating conditions (19). However, residues 525–530 approached the +0.2 threshold for nascent structure, indicating a mild helical tendency that, to our knowledge, has not been clearly documented for any RHIM protein in solution. Because ncSSP can underestimate sparsely populated ordered states due to ensemble averaging, we hypothesize that this local propensity may reflect a structurally relevant region and therefore explored complementary approaches to further probe it.

### Structural prediction suggests helix-prone regions flanking the RIPK1 RHIM

We next asked whether the 525–530 region exhibiting subtle conformational propensity in our NMR data would also be suggested by structure prediction. We therefore used AlphaFold3 (21) to model the N545D construct in an unassembled context.

**Figure 2:**
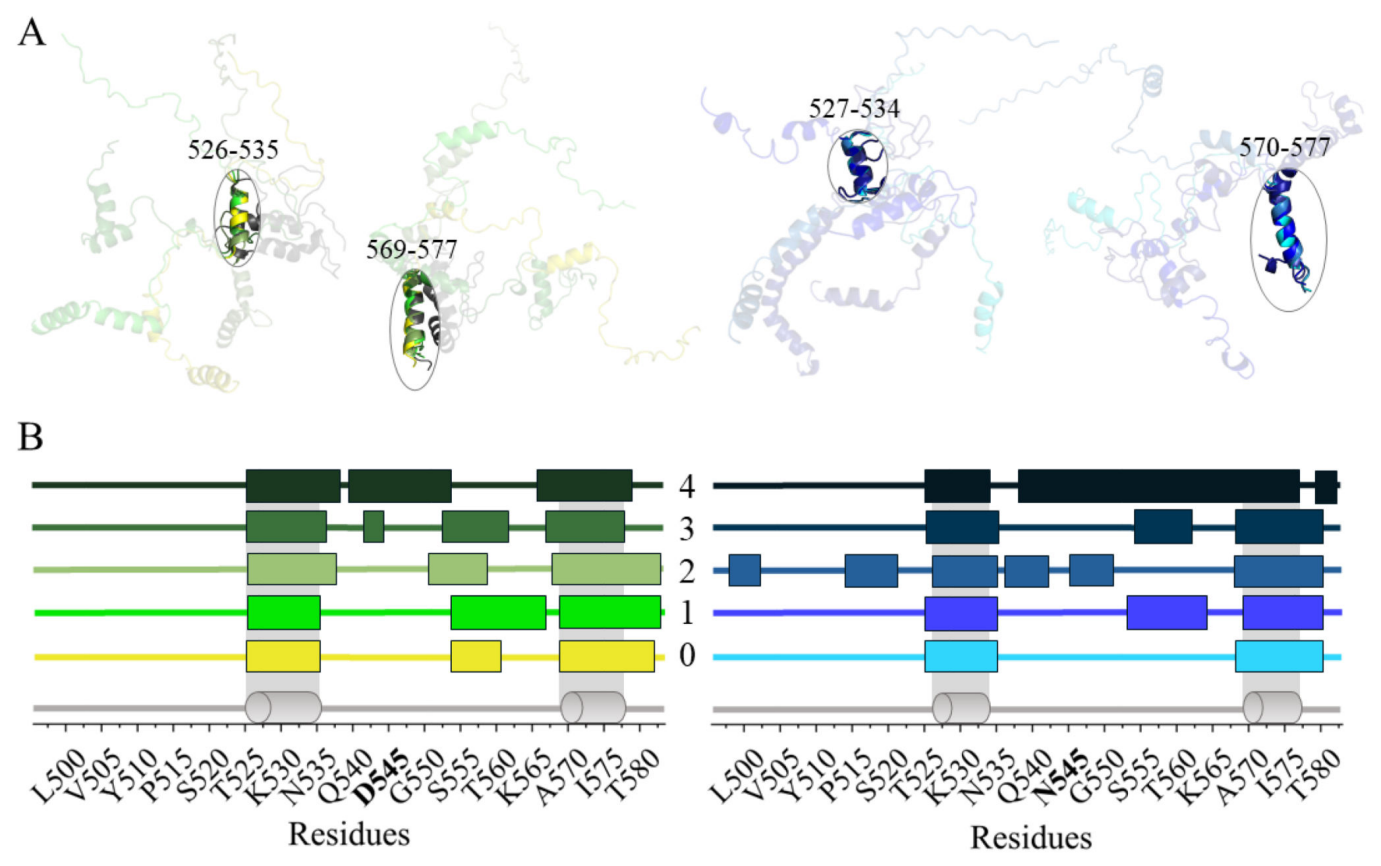
AlphaFold3 top-5 structural predictions for RIPK1 (residues 496–583), comparing the N545D mutant (green) and wild-type (blue). (**A**) Superposition of the five predicted models, aligned using the first (residues 526/527–534/535) and second (residues 569/570–577) consensus helical regions. (**B**) Schematic representation of the predicted secondary structure for each model, with disordered regions shown as flat lines and helical segments represented as colored boxes. The schematic consensus model is shown in grey, with consistent helices represented as cylinders and their positions highlighted by a light grey shaded area in the diagram.

AlphaFold3 generated five structural models (Figures 2A and 2B), with per-residue confidence scores (pLDDT) mostly below 70 and frequently below 50, indicating low to very low confidence. Nonetheless, the top-ranked models consistently suggested a largely disordered region encompassing the RHIM tetrad, flanked by short helix-like segments. Two helix-like stretches (526–535 and 569–577) recurred across all five models, with the former overlapping the region displaying the highest ncSSP scores.

To assess whether N545D influenced predictions, we repeated the analysis using the WT construct. The top five AlphaFold3 models again yielded low overall ranking confidence scores (0.66–0.32), while consistently suggesting a mainly disordered conformation with the same two recurrent helix-like segments (526–535 and 570–577). Altogether, AlphaFold3 suggests helix-prone regions flanking a disordered IQIG core motif in both WT and N545D constructs, motivating experimental validation.

### Micelles stabilize helical propensities and suppress aggregation in RIPK1 RHIM

We next assessed whether micelles could stabilize secondary-structure propensities in RIPK1 RHIM. A His-tagged N545D construct was produced as uniformly 13C,15N-labelled protein and purified in the presence of SDS above its critical micelle concentration. Buffer exchange into 0.5% SDS, 50 mM Tris, 5% D2O, pH 7.6 provided suitable conditions for NMR.

**Figure 3:**
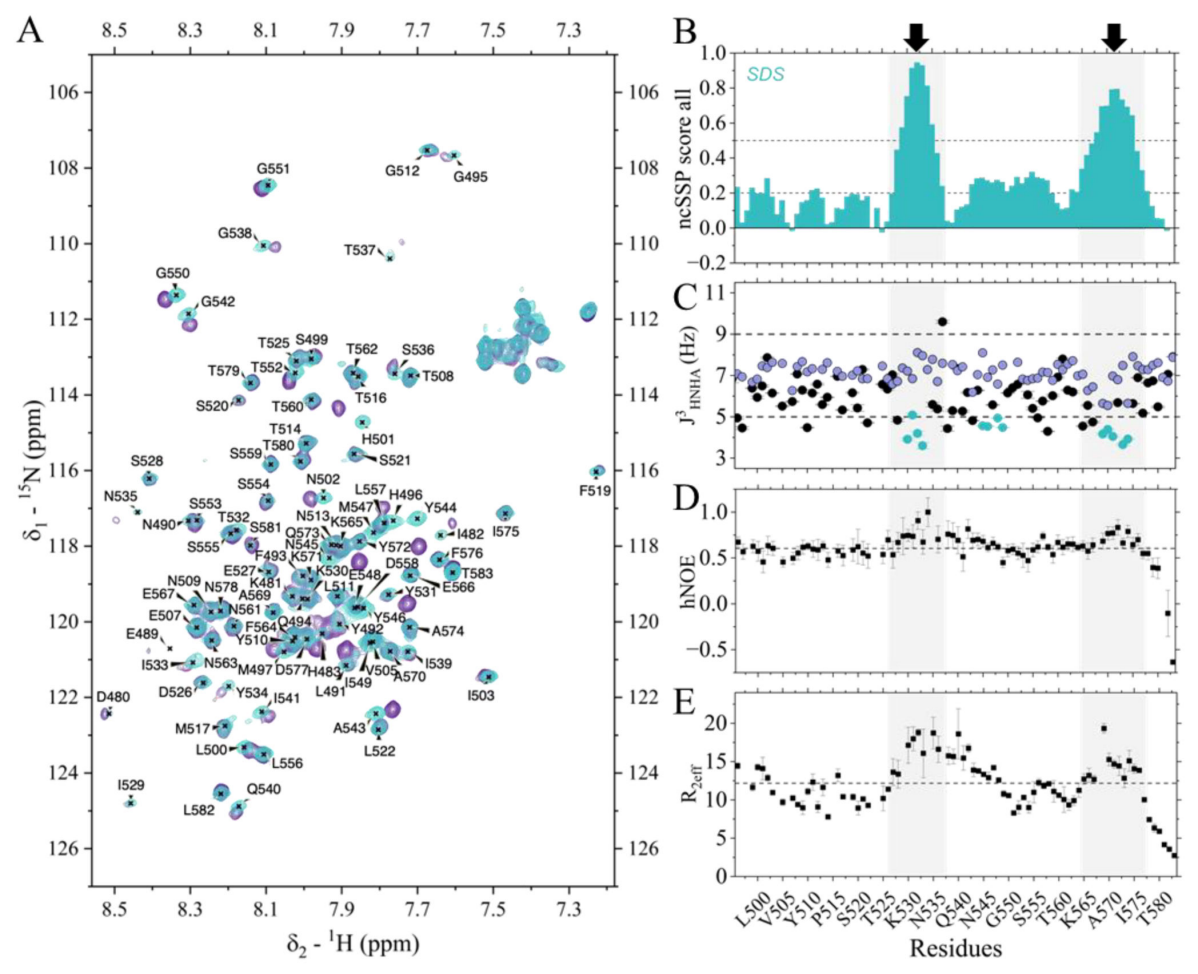
NMR characterization of RIPK1 WT RHIM in 0.5% SDS, 50 mM Tris, pH 7.6. **A)** Assigned ^1^H,^15^N-HSQC spectrum of WT (cyan) overlaid with the corresponding spectrum of N545D mutant (purple), both acquired under identical conditions. (**B**) Residue-specific ncSSP scores; dashed lines indicate reference thresholds for nascent (±0.2) and significant (±0.5) structural propensities. (**C**) Comparison of experimental (black) and random-coil predicted (lavender) ^3^J_HNHα_. Thresholds characteristic of β-sheet (~9 Hz) and α-helix (~5 Hz) conformations are indicated by dashed lines. Regions with consistent conformational tendencies (≥2 consecutive residues) are shown in blue. (**D**) hNOE values; mean shown as a dashed line. (**E**) *R*_2_,eff values; mean shown as a dashed line. Correlated trends across panels **B–E** are shaded in light grey and indicated with black arrows at the top.

The 1H,15N-HSQC spectrum displayed a markedly different fingerprint compared to aqueous buffer, spanning a broader chemical shift range (Figure 3A). 3D experiments enabled resonance assignment and chemical shift determination, from which ncSSP scores were calculated. Under micellar conditions, RIPK1 N545D RHIM exhibited increased ncSSP values relative to aqueous buffer (Figure S1) and revealed two segments with high α-helical propensity.

Encouraged by previous studies showing that SDS micelles prevent aggregation of RIPK3 RHIM (19), we next recorded analogous experiments on the WT construct under micellar conditions. We obtained a uniformly ^13^C,^15^N-labelled WT sample at 290 μM in 0.5% SDS, 50 mM Tris, 5% D2O, pH 7.6. The sample exhibited no significant signal decay over 3 days (Figure S2), consistent with suppressed aggregation. The ^1^H,^15^N-HSQC spectrum of the WT overlapped closely with that of the mutant, with differences confined to the mutated position and neighboring residues (Figure 3A). A 3D set yielded backbone and side chain assignments. Cα resonances were fully assigned except for P523 within a Pro–Pro repeat. Ser554 and Ser555 could not be distinguished due to a three-serine repeat, but this ambiguity did not affect subsequent analysis.

The ncSSP profile of WT closely matched that of N545D, indicating that the helical propensities observed under micellar conditions are not driven by the mutation (Figure 3B). Two stretches (527–537 and 563–577) exhibited ncSSP scores between 0.5 and 0.9, consistent with α-helical propensity and in agreement with AlphaFold3 predictions. The first stretch includes residues already showing mild helical bias in aqueous buffer, whereas the IQIG motif exhibited ncSSP values near zero, consistent with disorder.

### Micellar conditions reveal dynamically restricted segments in RIPK1 RHIM

To probe conformational dynamics under micellar conditions, we performed NMR experiments reporting on backbone flexibility. HNHA and HBHA(CO)NH experiments enabled determination of ^3^J_HNHα_ scalar couplings (22), which are sensitive to backbone dihedral angles. Typically, ^3^J_HNHα_ values below ~5 Hz are consistent with α-helical conformations, whereas values above ~9 Hz are characteristic of β-sheet conformations. Regions where conformational tendencies were observed for two or more consecutive residues are highlighted in blue in Figure 3C. Measured J-couplings deviated from random coil predictions (23), and residues 530–533 and 569–571 showed values consistent with α-helical propensity, agreeing with ncSSP trends for the 527–537 and 563–577 segments.

To obtain complementary information on backbone mobility, we measured {^1^H}–^15^N heteronuclear NOE (hNOE), *R*_1_ρ and *R*_1_ relaxation parameters. The hNOE profile revealed two regions spanning residues 528–542 and 569–576 that stood out from the average, consistent with increased rigidity (Figure 3D). Effective *R*_2_ (*R*_2_,eff) values derived from *R*_1_ and *R*_1_ρ measurements (24) were elevated across residues 527–546, indicating reduced internal mobility relative to flanking regions (Figure 3E). A similar restriction was evident for residues 569–576. In agreement, *R*_1_ values were below average for both segments (Figure S3).

Interestingly, relaxation measurements revealed an additional subsegment within the first region (approximately residues 537–546), which includes the conserved IQIG tetrapeptide, exhibiting elevated hNOE and *R*_2_,eff values (Figures 3D and 3E). Despite lacking defined secondary structure by ncSSP and ^3^J_HNHα_, this segment displayed relaxation behavior comparable to the preceding helical stretch (527–537). Notably, it spans the conserved IQIG tetrad and lies between the two helical regions, suggesting a dynamically restricted tether connecting these segments.

## Discussion

**Figure 4:**
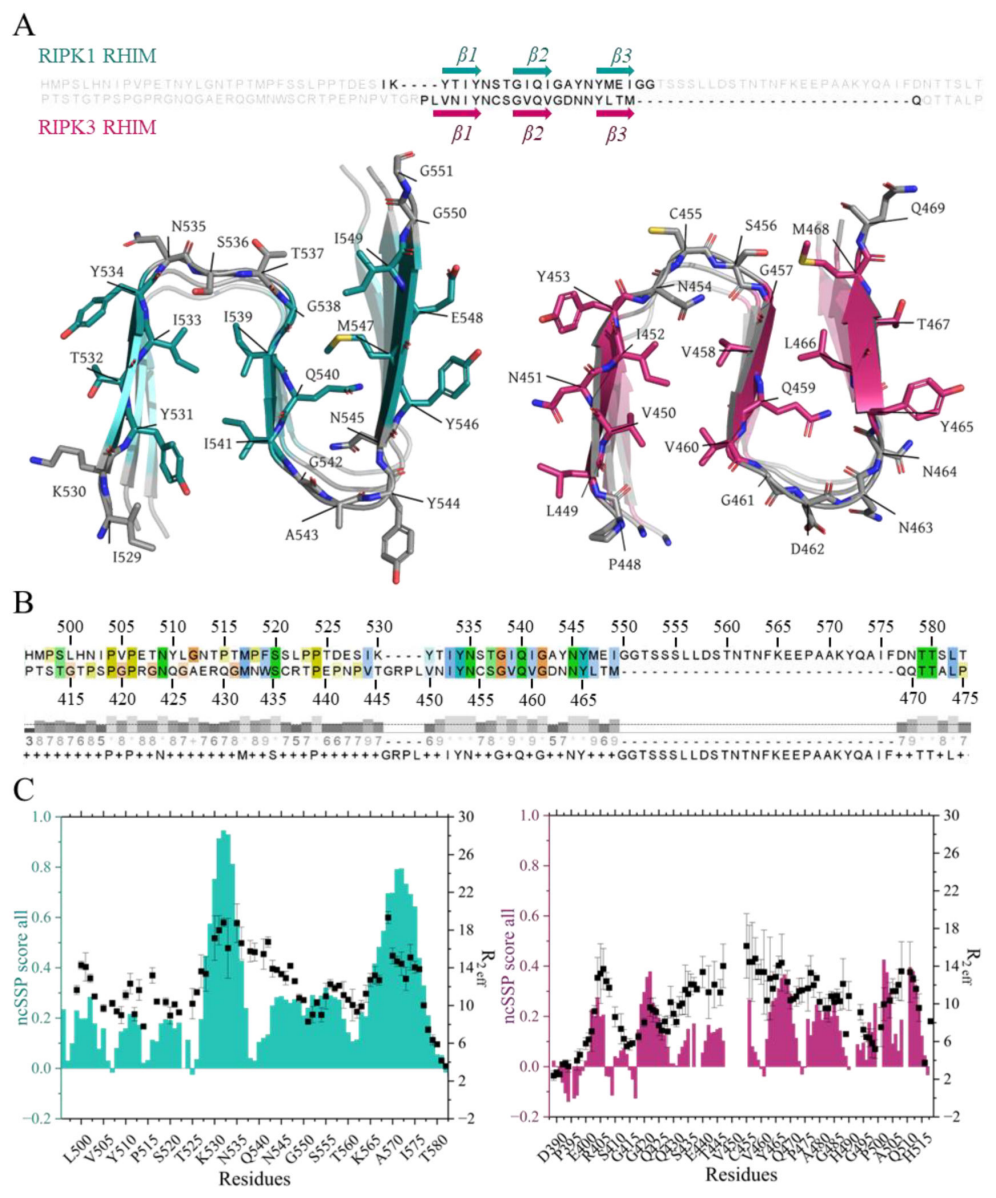
(**A**) CryoEM structures of amyloid fibrils formed by RIPK1 RHIM in cyan (9HR6) and RIPK3 RHIM in magenta (7DA4). At the top, the aligned sequences of RIPK1 (residues 496–583) and RIPK3 (residues 411–475) highlight in black the residues that form the fibril core resolved in the final structural models. (**B**) T-Coffee sequence alignment of RIPK1 (residues 496–583) and RIPK3 (residues 411–475) colored in Clustal, together with the alignment quality and consensus sequence. (**C**) Combined plots showing neighbor-corrected secondary structure propensities as columns and *R*_2_,eff relaxation rates as squares, for both RIPK1 (residues 496–583) in cyan and RIPK3 (residues 387–518) in magenta.

Due to challenges in purification and handling, few RHIM-containing proteins have been structurally characterized to date. Among experimentally studied human RHIMs—RIPK1, RIPK3, TRIF and ZBP1—only the assembled amyloid structures of RIPK1 and RIPK3 RHIMs have been resolved (10–13). In these structures, each protomer adopts a compact, N-shaped fold composed of three antiparallel β-strands, which assemble into tightly packed β-sheets stabilized by diverse molecular interactions (Figure 4A) (10,12,13).

The resemblance between their amyloid structures reflects the sequence similarity shared by RIPK1 and RIPK3 RHIMs. Sequence alignment shows notable homology, particularly within the conserved RHIM tetrapeptide (IQIG in RIPK1 and VQVG in RIPK3), which constitutes the central β-strand in the amyloid fold. Although variations occur in flanking regions, both sequences contain multiple conserved hydrophobic and aromatic residues, as well as numerous prolines (Figure 4B).

Given these similarities, one might anticipate comparable behavior for pre-assembly states. However, pre-fibrillar information reported for RIPK3 RHIM in micellar conditions showed no clear ordered conformation with ncSSP scores above 0.5 (18). This contrasts with our findings for RIPK1 RHIM, where micellar conditions reveal two distinct regions of high helical propensity (527–537 and 563–577) with ncSSP scores above 0.5 (Figure 4C), whereas analogous conditions do not elicit comparably populated helical states in RIPK3. Importantly, both RHIMs display relaxation profiles with elevated rates around the conserved tetrad and preceding residues, suggesting that micellar conditions impose a shared dynamic signature near the core motif that may contribute to aggregation suppression (Figure 4C) (18).

This differential pre-amyloid state, where RIPK1 populates more ordered helical conformations, may relate to its role as an initiator of necrosome assembly, enabling recruitment of RIPK3 and subsequent growth of RIPK3-rich assemblies (25,26). Given the intrinsic tendency to aggregate, RHIM exposure is likely tightly controlled in cells and restricted to conditions where signaling requires assembly. Conformational changes within the interdomain region of RIPK1 may contribute to RHIM accessibility and engagement in downstream signaling (6,27). Previous studies have described amyloid systems in which α-helical conformations precede β-sheet formation (28–34). In this context, we speculate that the helix-prone regions of RIPK1 RHIM—suggested with low confidence by AlphaFold3 and experimentally characterized here—could represent transient intermediates that facilitate conversion into the β-sheet architecture observed in assembled fibrils. While further work will be required to test this possibility directly, distinct pre-assembly conformational biases in RIPK1 versus RIPK3 provide a potential framework for understanding how RHIM assembly pathways may be regulated.

## Materials and methods

### Protein production and purification

Constructs encoding WT and N545D variants of RIPK1 RHIM (residues 496–583) were synthesized and cloned into a pET11a vector by GenScript, incorporating an N-terminal His×6 tag and codon optimization for E. coli expression. Plasmids were transformed into E. coli BL21 Star (DE3) cells and expressed in media supplemented with isotopically labeled carbon and nitrogen sources. Protein expression followed adapted protocols (35,36). Cells were grown at 37°C in LB medium to OD600 0.6, harvested, resuspended in M9 minimal medium containing 13C-D-glucose and 15NH4Cl (Cambridge Isotope Laboratories), and incubated for 1 h at 25°C. Expression was induced with 0.5 mM IPTG overnight. Cells were resuspended in lysis buffer (50 mM Tris, 300 mM NaCl, 1 μg/mL DNase I) and lysed by sonication on ice. Debris was removed by centrifugation, yielding inclusion bodies.

For purification under near-physiological conditions, inclusion bodies were solubilized in 8 M urea, 150 mM NaCl, 50 mM Tris, 1 mM DTT, pH 8.0, sonicated, and clarified by centrifugation. The supernatant was loaded onto a HisTrap column (Cytiva), washed, and eluted with 0.5 M imidazole. Final buffer exchange into 20 mM MES, 5% D2O, pH 6.6 was performed using a PD-10 MidiTrap column (Cytiva).

For purification under micellar conditions, inclusion bodies were solubilized in 1% SDS, 150 mM NaCl, 50 mM Tris, 1 mM DTT, pH 8.0, sonicated, clarified, and loaded onto a HisTrap column. The column was washed in 1% SDS and then 0.5% SDS. Elution used 0.5% SDS supplemented with 0.5 M imidazole. Final NMR buffer was 0.5% SDS, 50 mM Tris, 5% D2O, pH 7.6 (PD-10 MidiTrap).

### NMR experiments

All NMR experiments were conducted at 298 K on a Bruker Avance Neo 800 MHz spectrometer (^1^H frequency), equipped with a TCI cryoprobe and Z-gradients. For N545D assignment in 20 mM MES, 5% D2O, pH 6.6, 1H-15N HSQC, H(NCOCA)HN, (H)N(COCA)NH, HNCA, CBCA(CO)NH and (H)CC(CO)NH were collected on a ~21 μM sample. For N545D in micelles, CA, N and H chemical shifts were obtained from 1H-15N HSQC and HNCA on a ~53 μM sample. For WT in 0.5% SDS, 50 mM Tris, 5% D2O, pH 7.6, ^1^H-^15^N HSQC, H(NCOCA)HN, (H)N(COCA)NH, HNCA, CBCA(CO)NH, HNCO, HN(CA)CO, HNCACB and (H)CC(CO)NH were collected on a ~290 μM sample. Table S1 contains all the experimental details to reproduce these experiments.

J couplings were derived from HNHA and HBHA(CO)NH (22). hNOE values were calculated from interleaved saturated/unsaturated spectra. ^15^N-*R*_1_ and ^15^N-*R*_1_ρ experiments used standard Bruker sequences with the delays specified in Table S1; relaxation curves were fitted with Dynamics Centre. Spectra were processed in Topspin 4.4.1 (Bruker). Data were apodized with QSIN (SSB=2) in all dimensions. For 2D spectra, LB=0.3 Hz was applied; for 3D spectra LB was 1 Hz (F3), 0.3 Hz (F2), 0 Hz (F1). Zero-filling was applied in indirect dimensions. Chemical shifts were referenced to TMS. Spectra were analyzed with CCPN Analysis 3 (37) and POKY (38). NMR data are deposited in the BMRB with IDs 53401 and 53411.

### Structural predictions and analysis

Predictions were performed with AlphaFold3 (21). ncSSP values were calculated using the Mulder lab webserver and the Tamiola reference library (20). Predicted random-coil J couplings were generated using the Bax group webserver (23). ^15^N-*R*_2_,eff values were obtained from *R*_1_ and *R*_1_ρ following Palmer and Massi definitions (24). Sequence alignment used Jalview with T-Coffee and Clustal coloring; only residues 496–583 of RIPK1 and 411–475 of RIPK3 are shown.

## Supplementary Material

Supporting information

## Highlights

- Solution NMR access to RIPK1 RHIM pre-assembly states is enabled by a structure-guided, slow-aggregating N545D variant at near-physiological pH.
- SDS micelles suppress RIPK1 RHIM aggregation and stabilize two α-helical propensities flanking the disordered IQIG core (~527–537 and ~563–577), enabling measurements on wild-type sequence.
- Micelle-stabilized helical tendencies are supported by chemical-shift–based ncSSP and backbone reporters (J-couplings/hNOE/R2eff).
- Under analogous micellar conditions, RIPK3 does not show comparable α-helical populations, suggesting distinct pre-assembly conformational biases despite conserved RHIM amyloid endpoints.

## References

[1] Xie T, Peng W, Liu Y, Yan C, Maki J, Degterev A, Yuan J, Shi Y (2013). Structural basis of RIP1 inhibition by necrostatins. Structure.

[2] Ding J, Pan X, Du L, Yao Q, Xue J, Yao H, Wang D-C, Li S, Shao F (2019). Structural and functional insights into host death domains inactivation by the bacterial arginine GlcNAcyltransferase effector. Mol Cell.

[3] Li J, McQuade T, Siemer AB, Napetschnig J, Moriwaki K, Hsiao Y-S, Damko E, Moquin D, Walz T, McDermott A (2012). The RIP1/RIP3 necrosome forms a functional amyloid signaling complex required for programmed necrosis. Cell.

[4] Meng H, Liu Z, Li X, Wang H, Jin T, Wu G, Shan B, Christofferson DE, Qi C, Yu Q (2018). Death-domain dimerization-mediated activation of RIPK1 controls necroptosis and RIPK1-dependent apoptosis. Proc Natl Acad Sci USA.

[5] Newton K, Wickliffe KE, Maltzman A, Dugger DL, Strasser A, Pham VC, Lill JR, Roose-Girma M, Warming S, Solon M (2016). RIPK1 inhibits ZBP1-driven necroptosis during development. Nature.

[6] Orozco S, Yatim N, Werner MR, Tran H, Gunja SY, Tait SW, Albert ML, Green DR, Oberst A (2014). RIPK1 both positively and negatively regulates RIPK3 oligomerization and necroptosis. Cell Death Differ.

[7] Pham CL, Shanmugam N, Strange M, O’Carroll A, Brown JW, Sierecki E, Gambin Y, Steain M, Sunde M (2019). Viral M45 and necroptosis-associated proteins form heteromeric amyloid assemblies. EMBO Rep.

[8] Riebeling T, Kunzendorf U, Krautwald S (2022). The role of RHIM in necroptosis. Biochem Soc Trans.

[9] Mompeán M, Li W, Li J, Laage S, Siemer AB, Bozkurt G, Wu H, McDermott AE (2018). The structure of the necrosome RIPK1-RIPK3 core, a human hetero-amyloid signaling complex. Cell.

[10] Wu X, Ma Y, Zhao K, Zhang J, Sun Y, Li Y, Dong X, Hu H, Liu J, Wang J (2021). The structure of a minimum amyloid fibril core formed by necroptosis-mediating RHIM of human RIPK3. Proc Natl Acad Sci USA.

[11] Ma Y, Zhang Q, Li D, Zhao K, Li Z, Liu Y, Wang C, Sun B, Li D, Yuan J, Liu C (2025). Intercellular propagation of RIPK1/RIPK3 amyloid fibrils. Proc Natl Acad Sci USA.

[12] Polonio P, López-Alonso JP, Jiang H, Escobedo F, Titaux G, Ubarretxena-Belandia I, Mompeán M (2025). Structural basis for the assembly of amyloid fibrils by the master cell-signaling regulator human receptor-interacting protein kinase 1. Nat Commun.

[13] He C, Varghese NR, Keeler EG, Pham CLL, Williams B, Tetter S, Semaan C, Wilde KL, Brown SHJ, Bouwer JC (2025). Structural studies of an anti-necroptosis viral:human functional hetero-amyloid M45:RIPK3 using SSNMR. bioRxiv.

[14] Sawada M, Yamaguchi K, Hirano M, Noji M, So M, Otzen D, Kawata Y, Goto Y (2020). Amyloid formation of α-synuclein based on the solubility- and supersaturation-dependent mechanism. Langmuir.

[15] So M, Ishii A, Hata Y, Yagi H, Naiki H, Goto Y (2015). Supersaturation-limited and unlimited phase spaces compete to produce maximal amyloid fibrillation near the critical micelle concentration of sodium dodecyl sulfate. Langmuir.

[16] Williamson JA, Loria JP, Miranker AD (2009). Helix stabilization precedes aqueous and bilayer-catalyzed fiber formation in islet amyloid polypeptide. J Mol Biol.

[17] Zhang L, Kang H, Vázquez FX, Toledo-Sherman L, Luan B, Zhou R (2017). Molecular mechanism of stabilizing the helical structure of huntingtin N17 in a micellar environment. J Phys Chem B.

[18] Escobedo-González FC, Gelardo A, Reimers A, Polonio P, Mompeán M, Titaux-Delgado GA (2025). Membrane charge primes the necroptotic kinase RIPK3 for amyloid assembly. Commun Chem.

[19] Pham CLL, Titaux-Delgado GA, Varghese NR, Polonio P, Wilde KL, Sunde M, Mompeán M (2023). NMR characterization of an assembling RHIM (RIP homotypic interaction motif) amyloid reveals a cryptic region for self-recognition. J Biol Chem.

[20] Tamiola K, Acar B, Mulder FAA (2010). Sequence-specific random coil chemical shifts of intrinsically disordered proteins. J Am Chem Soc.

[21] Abramson J, Adler J, Dunger J, Evans R, Green T, Pritzel A, Ronneberger O, Willmore L, Ballard AJ, Bambrick J (2024). Accurate structure prediction of biomolecular interactions with AlphaFold 3. Nature.

[22] Vuister GW, Bax A (1993). Quantitative J correlation: A new approach for measuring homonuclear three-bond J(HN–Hα) coupling constants in 15N-enriched proteins. J Am Chem Soc.

[23] Shen Y, Roche J, Grishaev A, Bax A (2018). Prediction of nearest neighbor effects on backbone torsion angles and NMR scalar coupling constants in disordered proteins. Protein Sci.

[24] Palmer AG, Massi F (2006). Characterization of the dynamics of biomacromolecules using rotating-frame spin relaxation NMR spectroscopy. Chem Rev.

[25] Amusan OT, Wang S, Yin C, Koehler HS, Li Y, Tenev T, Wilson R, Bellenie B, Zhang T, Wang J (2025). RIPK1 is required for ZBP1-driven necroptosis in human cells. PLoS Biol.

[26] Nan N, Hu H, Zhu X, Liu J, Yuan F, Li Z, Wang H (2025). RIPK1 S213E mutant suppresses RIPK1-dependent cell death by preventing interactions with RIPK3 and CASP8. Cell Death Discov.

[27] Delanghe T, Dondelinger Y, Bertrand MJM (2020). RIPK1 kinase-dependent death: a symphony of phosphorylation events. Trends Cell Biol.

[28] Anderson VL, Ramlall TF, Rospigliosi CC, Webb WW, Eliezer D (2010). Identification of a helical intermediate in trifluoroethanol-induced alpha-synuclein aggregation. Proc Natl Acad Sci USA.

[29] Asakura T, Nishimura A, Sato Y (2017). Quantitative correlation between primary sequences and conformations in 13C-labeled *Samia cynthia ricini* silk fibroin during strain-induced conformational transition by 13C solid-state NMR. Macromolecules.

[30] De Vocht ML, Reviakine I, Ulrich W, Bergsma-Schutter W, Wösten HAB, Vogel H, Brisson A, Wessels JGH, Robillard GT (2002). Self-assembly of the hydrophobin SC3 proceeds via two structural intermediates. Protein Sci.

[31] Fezoui Y, Teplow DB (2002). Kinetic studies of amyloid β-protein fibril assembly. J Biol Chem.

[32] Jiang L-L, Che M-X, Zhao J, Zhou C-J, Xie M-Y, Li H-Y, He J-H, Hu H-Y (2013). Structural transformation of the amyloidogenic core region of TDP-43 protein initiates its aggregation and cytoplasmic inclusion. J Biol Chem.

[33] Kallberg Y, Gustafsson M, Persson B, Thyberg J, Johansson J (2001). Prediction of amyloid fibril-forming proteins. J Biol Chem.

[34] Kirkitadze MD, Condron MM, Teplow DB (2001). Identification and characterization of key kinetic intermediates in amyloid β-protein fibrillogenesis. J Mol Biol.

[35] Marley J, Lu M, Bracken C (2001). A method for efficient isotopic labeling of recombinant proteins. J Biomol NMR.

[36] Sivashanmugam A, Murray V, Cui C, Zhang Y, Wang J, Li Q (2009). Practical protocols for production of very high yields of recombinant proteins using *Escherichia coli*. Protein Sci.

[37] Skinner SP, Fogh RH, Boucher W, Ragan TJ, Mureddu LG, Vuister GW (2016). CcpNmr AnalysisAssign: a flexible platform for integrated NMR analysis. J Biomol NMR.

[38] Lee W, Rahimi M, Lee Y, Chiu A (2021). POKY: a software suite for multidimensional NMR and 3D structure calculation of biomolecules. Bioinformatics.

