# Supplementary material for: Solution NMR reveals micelle-stabilized α-helical segments flanking the RIPK1 RHIM amyloid core
Source: Biochem Biophys Res Commun. Author manuscript; Available in PMC 2026 Apr 16. (PMC7618886; doi:10.1016/j.bbrc.2026.153513)
Supplement: Supporting information [file EMS212681-supplement-Supporting_information.docx]

# ***Supporting Information for:***

**Solution NMR reveals micelle-stabilized α-helical segments flanking the RIPK1 RHIM amyloid core**

Paula Polonio^1,2^, Gustavo A. Titaux-Delgado^1^, Miguel Mompeán^1*^

^1^ Instituto de Química Física Blas-Cabrera (IQF-CSIC), Madrid, Spain

^2^ Universidad Autónoma de Madrid, Escuela de Doctorado, Madrid, Spain

*Correspondence: Miguel Mompeán, [mmompean@iqf.csic.es](mailto:mmompean@iqf.csic.es)

**Figure S1.** Neighbour-corrected secondary structure propensity (ncSSP) scores for RIPK1 N545D RHIM in **A)** 0.5% SDS, 50 mM Tris, pH 7.6 and **B)** 0.5% SDS, 50 mM Tris, pH 7.6 overlapped with 20 mM MES, pH 6.6. Dashed lines indicate threshold values for nascent structure (±0.2) and notable structural propensity (±0.5).





**Figure S2.** Normalized intensity decay for ^1^H,^15^N-HSQC spectra of RIPK1 WT RHIM in 0.5% SDS, 50 mM Tris, pH 7.6, along 3 days. Residues corresponding to prolines, which lack amide signals, are indicated in dark grey.





**Figure S3.** Combined plot showing the opposite trend followed by RIPK1 WT RHIM in 0.5% SDS, 50 mM Tris, pH 7.6 relaxation rates. In black (left Y axis), R2 effective rates; in cyan (right Y axis), R1 rates; shaded in light grey conformational propensities consistent with ncSSPs, J-coupling constants and hNOE data.

**Table S1.** Experimental details for the different collected NMR spectra.

| Sample | Experiment | NS | TD | SW (ppm) | Carrier (ppm) | SI |
| --- | --- | --- | --- | --- | --- | --- |
| N545D in MES, pH 6.6 | ^1^H-^15^N HSQC | 8 | 256(^15^N) | 12(^1^H); 20(^15^N) | 4.7(^1^H); 116(^15^N) | 512(^15^N) |
|  | H(NCOCA)HN | 60 | 64(^15^N); 64(^1^H) | 12(^1^H); 20(^15^N); 4.8(^1^H) | 4.7(^1^H); 116(^15^N); 4.7(^1^H) | 128(^15^N); 128(^1^H) |
|  | (H)N(COCA)NH | 60 | 64(^15^N); 64(^15^N) | 12(^1^H); 20(^15^N); 20(^15^N) | 4.7(^1^H); 116(^15^N); 116(^15^N) | 128(^15^N); 128(^15^N) |
|  | HNCA | 24 | 62(^15^N); 94(^13^C) | 12(^1^H); 20(^15^N); 25(^13^C) | 4.7(^1^H); 116(^15^N); 52.5(^13^C) | 128(^15^N); 256(^13^C) |
|  | CBCA(CO)NH | 20 | 64(^15^N); 96(^13^C) | 12(^1^H); 20(^15^N); 80(^13^C) | 4.7(^1^H); 116(^15^N); 43(^13^C) | 128(^15^N); 256(^13^C) |
|  | (H)CC(CO)NH | 32 | 54(^15^N); 80(^13^C) | 12(^1^H); 20(^15^N); 80(^13^C) | 4.7(^1^H); 116(^15^N); 43(^13^C) | 128(^15^N); 256(^13^C) |
| N545D in SDS, pH 7.6 | ^1^H-^15^N HSQC | 8 | 156(^15^N) | 12(^1^H); 20(^15^N) | 4.7(^1^H); 116(^15^N) | 512(^15^N) |
|  | HNCA | 40 | 62(^15^N); 94(^13^C) | 12(^1^H); 20(^15^N); 25(^13^C) | 4.7(^1^H); 116(^15^N); 52.5(^13^C) | 128(^15^N); 256(^13^C) |
| WT in SDS, pH 7.6 | ^1^H-^15^N HSQC | 8 | 512(^15^N) | 12(^1^H); 20(^15^N) | 4.7(^1^H); 116(^15^N) | 2048(^15^N) |
|  | H(NCOCA)HN | 32 | 64(^15^N); 64(^1^H) | 12(^1^H); 20(^15^N); 4.8(^1^H) | 4.7(^1^H); 116(^15^N); 4.7(^1^H) | 128(^15^N); 128(^1^H) |
|  | (H)N(COCA)NH | 32 | 64(^15^N); 64(^1^N) | 12(^1^H); 20(^15^N); 20(^15^N) | 4.7(^1^H); 116(^15^N); 116(^15^N) | 128(^15^N); 128(^15^N) |
|  | HNCA | 16 | 64(^15^N); 94(^13^C) | 12(^1^H); 20(^15^N); 25(^13^C) | 4.7(^1^H); 116(^15^N); 52.5(^13^C) | 128(^15^N); 256(^13^C) |
|  | CBCA(CO)NH | 20 | 64(^15^N); 94(^13^C) | 12(^1^H); 20(^15^N); 80(^13^C) | 4.7(^1^H); 116(^15^N); 43(^13^C) | 128(^15^N); 256(^13^C) |
|  | HNCO | 8 | 60(^15^N); 128(^13^C) | 12(^1^H); 20(^15^N); 14(^13^C) | 4.7(^1^H); 116(^15^N); 173.5(^13^C) | 128(^15^N); 256(^13^C) |
|  | HN(CA)CO | 24 | 60(^15^N); 104(^13^C) | 12(^1^H); 20(^15^N); 14(^13^C) | 4.7(^1^H); 116(^15^N); 173.5(^13^C) | 128(^15^N); 256(^13^C) |
|  | HNCACB | 56 | 64(^15^N); 112(^13^C) | 12(^1^H); 20(^15^N); 80 (^13^C) | 4.7(^1^H); 116(^15^N); 39.5(^13^C) | 256 (^15^N); 512(^13^C) |
|  | (H)CC(CO)NH | 16 | 56(^15^N); 80(^13^C) | 12(^1^H); 20(^15^N); 80(^13^C) | 4.7(^1^H); 116(^15^N); 43(^13^C) | 128(^15^N); 256(^13^C) |
|  | HNHA | 16 | 128(^1^H); 54(^15^N) | 12(^1^H); 12(^1^H); 20(^15^N) | 4.7(^1^H); 4.7(^1^H); 116(^15^N) | 256(^1^H); 128(^15^N) |
|  | HBHA(CO)NH | 16 | 56(^15^N); 180(^1^H) | 12(^1^H); 20(^15^N) 6(^1^H) | 4.7(^1^H); 116(^15^N); 4.7(^1^H) | 256(^1^H); 128(^15^N) |
|  | hNOE | 16 | 196(^15^N) | 12(^1^H); 20(^15^N) | 4.7(^1^H); 116(^15^N) | 1024(^15^N) |
|  | *R*_1_ | 8 | 144(^15^N) | 12(^1^H); 20(^15^N) | 4.7(^1^H); 116(^15^N) | 512(^15^N) |
|  | *R*_1_ρ | 8 | 188(^15^N) | 12(^1^H); 20(^15^N) | 4.7(^1^H); 116(^15^N) | 512(^15^N) |
